# Effectiveness of a behaviour change intervention on health literacy for behavioural risk factors of non-communicable diseases among health care assistants of government hospitals in Colombo District: a cluster-randomized controlled trial

**DOI:** 10.1186/s12889-025-26110-9

**Published:** 2026-01-02

**Authors:** Irshad Mashood, Dulani Samaranayake, Vindya Kumarapeli

**Affiliations:** 1 NCD, Lifestyle, Social Media and Community Health Promotion unit, Health Promotion Bureau, Ministry of Health, Kynsey Road, Colombo 10, Colombo, Sri Lanka; 2 Department of Community Medicine, Faculty of Medicine, University of Colombo, Colombo 10, Colombo, Sri Lanka; 3 Specialist in Community Medicine, Directorate of Policy Analysis and Development, Ministry of Health, Colombo 10, Colombo, Sri Lanka; 4 38/1 A, Dematagoda place, Colombo 09, Colombo, Sri Lanka

**Keywords:** Health literacy, Behavioural risk factors of non-communicable diseases, Behaviour change intervention, Cluster-randomized controlled trial

## Abstract

**Background:**

Non-communicable diseases (NCDs) are a major public health challenge in Sri Lanka, driven by behavioural risk factors. Healthcare Assistants (HCAs) play a key role in NCD prevention, but limited health literacy (HL) reduces their effectiveness. Strengthening HL among HCAs can reduce their own NCD risk, enhance their role as health promoters, and improve patient education and hospital-based health initiatives.

**Objectives:**

This study aimed to assess the effectiveness of a behaviour change intervention package (BCIP) to improve HL on behavioural risk factors of NCDs among HCAs in Colombo district.

**Methods:**

A cluster Randomized Controlled Trial (cRCT) was conducted with 240 HCAs from 20 hospitals in the Colombo district, 10 hospitals each for the control and intervention arms. The 16 sessions of the BCIP were conducted over eight weeks. The HL-NCD tool and STEPS questionnaire were administered two weeks before and after the intervention. HL score was analyzed as a continuous variable, while HL level was categorized as inadequate or adequate based on predefined HL score cutoffs. Analyses included unadjusted, cluster-level, and Generalized Estimating Equations (GEE) models to account for clustering effects.

**Results:**

The intervention group showed significant improvements in HL score (unadjusted *p* < 0.001; cluster *p* = 0.005; GEE OR = 7.80, 95% CI: 7.38–8.22, *p* < 0.001) and HL level (unadjusted *p* = 0.003; cluster *p* = 0.011; GEE OR = 3.80, 95% CI: 1.59–9.11, *p* = 0.003). Age < 35 years was a significant positive predictor of HL score and educated only up to O/Ls was a significant negative predictor of HL level. The GEE analysis revealed significant improvement in behaviour change related to diet, physical activity, avoiding smokeless tobacco, and non-exposure to secondhand smoking except avoiding smoking tobacco and alcohol intake.

**Conclusions:**

The BCIP was effective in improving HL and reducing behavioural risk factors of NCDs among HCAs, highlighting its potential for broader implementation.

**Clinical trial number:**

SLCTR/2023/023, https://slctr.lk/trials/slctr-2023-023, 11th December 2023, Sri Lanka Clinical Trial Registry.

**Supplementary Information:**

The online version contains supplementary material available at 10.1186/s12889-025-26110-9.

## Introduction

Non-communicable diseases (NCDs) are the leading cause of premature death worldwide, primarily driven by behavioural risk factors such as unhealthy diet, physical inactivity, tobacco use, and alcohol intake [1]. Limited health literacy (HL) exacerbates NCD risk and worsens outcomes, making HL-based interventions crucial for prevention [2]. In Sri Lanka, 91.4% of adults exhibit at least one behavioural risk factor, including poor diet, high salt intake, low physical activity, tobacco use, alcohol consumption, and exposure to secondhand smoke. Despite slightly better dietary habits, overall adherence to healthy behaviours remains poor, highlighting the need for targeted behaviour-focused interventions [3].

HL extends beyond the ability to read and write health information. It encompasses the capacity to access, understand, appraise, and apply health information to make informed decisions that influence lifestyle and health outcomes [4, 5]. Theoretical models such as Nutbeam’s framework conceptualize HL as a continuum of functional, communicative, and critical competencies [6], while the Calgary Charter on Health Literacy defines HL as a dynamic process linking knowledge to behavioural action [7]. According to the Charter, behaviour change occurs through sequential processes of finding, understanding, evaluating, communicating, and using health information to improve health decisions and practices.

Improvements in HL are therefore expected to influence specific behavioural outcomes through enhanced knowledge, motivation, self-efficacy, and the ability to navigate and act on health information [8]. HL-based interventions, unlike traditional information campaigns, emphasize empowerment and skill-building, thereby producing more sustained changes in diet, physical activity, tobacco use, and alcohol consumption [9]. Evidence from global HL initiatives such as the Canyon Ranch Institute Life Enhancement Program (CRI-LEP) and the WHO National Health Literacy Demonstration Projects (NHLDPs) demonstrates the effectiveness of this approach in reducing behavioural risk factors and promoting self-care [10, 11].

Within Sri Lanka, HL research has predominantly focused on groups such as pregnant mothers, schoolteachers, and environmental officers, mainly assessing knowledge and attitudes rather than behaviour change capacity [12–14]. However, Healthcare Assistants (HCAs) represent a critical but overlooked occupational group. They not only experience a high prevalence of NCD-related behavioural risks but also interact daily with patients, giving them the potential to serve as role models and change agents within hospital settings [15]. Strengthening HL among HCAs could therefore reduce their personal risk, enhance patient education, and support institutional health promotion initiatives [16].

To address this gap, a Behaviour Change Intervention Package (BCIP**)** was developed, guided by constructs from the Calgary Charter each intervention session designed to enhance participants’ ability to find standard health information on NCD risk factors, understand their practice, compare their practice with the standard, and apply standard health information to their practice. The present study evaluates the effectiveness of this BCIP in improving HL and promoting positive behavioural changes among HCAs in the Colombo District, thereby contributing to both local and global evidence on HL-based interventions for NCD prevention.

## Methodology

### Study design

Cluster randomized controlled trial.

### Study setting

This study was conducted in 20 hospitals in Colombo district.

### Study population

HCAs working in government hospitals in Colombo district. 

Inclusion criteria: Permanent staff who have been working as HCA more than six months and age of HCA in between 18 years to 55 years were included.

Exclusion criteria: Staff who plan to retire or transfer within a year, HCAs who were on long leave e.g.: maternity leave or medical leave, HCAs who were detected as having NCD or facing complications like CKD and HCAs in PMCUs of Colombo district were excluded.

### Study period

Study period was from June 2023 to June 2024.

### Sample size

A cluster was defined as 12 HCAs selected from each hospital. The required number of clusters for this cluster-randomized controlled trial was calculated using the standard formula for comparing means, incorporating the design effect to account for intra-cluster correlation.

$$\begin{array}{c}C=1+\left(Z_{\alpha/2}\right)^2\left(\sigma_{0^2}+\sigma_{1^2}\right)\times\\\;\left[1+\left(m-1\right)\rho\right]/\mathrm m\left(\mu_0-\mu_1\right)^2\end{array}$$

Sample size was based on effect sizes from a previous Iranian study where an intervention markedly increased S-TOFHLA scores in diabetic patients. A cluster size of 12 and ICC of 0.04 were used based on a CRT on health communication for diabetic patients.

C = Number of clusters.

Z_α/2_ = The standard normal deviate for α, 0.05 = 1.96.

Z_β_ = The standard normal deviate for β, 0.2 = 0.84.

m = Size of the cluster = 12.

µ_0_ = True mean of outcome variable in the intervention group = 132.14.

µ_1_ = True mean of outcome variable in the control group = 94.33.

σ_0_ = Standard deviation of outcome variable in the intervention group = 13.81.

σ_1_ = Standard deviation of outcome variable in the control group = 12.64.

ρ = Intraclass correlation coefficient (ICC) = 0.04.

$$\begin{aligned} \mathrm C&=1+\left(1.96+0.84\right)^2\left(13.812+12.642\right)\;\times\;\\&\left[1+\left(12.1\right)0.04\right]\;/\;12\left(132.14-94.33\right)^2\end{aligned}$$

C = 9.7 Number of clusters per arm was taken as 10.

Substituting these values yielded approximately 9.7 clusters per arm; therefore, 10 clusters (hospitals) were included in each arm, giving a total of 20 clusters and 240 participants. No attrition occurred during the study, as all recruited participants completed both baseline and post-intervention assessments.

### Sampling technique

Stratified cluster sampling method was applied according to the type of hospitals in Colombo district.

Stratification **-** Types of hospitals in Colombo district are Teaching hospitals, Special hospitals, Base hospitals and Divisional hospitals and these served as strata for the sampling. Number of clusters to be selected from each stratum was calculated proportionate to the number of HCAs in each of them. Each hospital was taken as cluster and there were twenty clusters (6 teaching hospitals, 4 special hospitals, 4 base hospitals, 6 divisional hospitals).

Recruitment of HCAs **-** Required number of HCAs was selected randomly from the pay roll of all HCAs attached to the hospital. It was selected in three steps within the hospital.

- Step I: All HCAs were listed out based on pay roll.
- Step II: Eligible HCAs were selected based on inclusion and exclusion criteria.
- Step III: Required number of HCAs were selected using simple random sampling (random table).

### Study instruments

I) HL-NCD Questionnaire - The primary outcome was the improvement in HL on behavioural risk factors of NCDs. The Health Literacy on Behavioural Risk Factors of NCDs (HL-NCD) tool (Supplementary File 01), developed and validated for HCAs in Sri Lanka, was used for assessment. It comprised 56 items under four factors: Finding Standard Information, Understanding Self-Practice, Comparing, and Applying explaining 92.49% of the variance. The tool showed excellent construct validity (CFI = 0.99, RMSEA = 0.0072, SRMR = 0.03) and strong reliability (Cronbach’s α = 0.901; ICC = 0.836), with significant correlations to key behavioural practices (ρ = 0.43–0.58).
  Section A – Included a set of questions to assess basic characteristics such as socio-demographic characteristics, work-related characteristics, language proficiency, source of obtaining information and level of health knowledge of the participants.
  Section B – Proper HL-NCD tool to assess HL of the participants related to behavioural risk factors of NCDs.
II) STEPS Questionnaire: Secondary outcomes were behaviour change related to pattern of Diet, frequency of physical activity, status/frequency of tobacco use and alcohol intake. These were assessed using the WHO STEPS questionnaire. The Sri Lankan version, previously validated and used in national surveys (2003–2021) to assess behavioural NCD risk factors, was employed. Relevant behavioural components CORE T (Tobacco use), CORE A (Alcohol intake), CORE D (Diet), and CORE P (Physical activity) were included.

### Data collection

Data collection was conducted by principal investigator (PI) and research assistants (RAs) who were trained on administering the questionnaire. The selected HCAs were met by the RAs in person as appointment and they were explained the purpose of the study and informed written consent was obtained. Then the questionnaires were administered to the participants at two time points: baseline assessment and post-assessment. Data collectors were blinded to the group allocation of participants only at baseline assessment.

### Study implementation

I) Baseline Assessment - Baseline assessment was conducted two weeks before the implementation of the BCIP. Participants and data collectors were not informed regarding the allocation of intervention and control arm at baseline. Data collection was conducted using tools: HL-NCD and selected components of STEPS questionnaire.
II) Randomization - Randomization by cluster was implemented in two steps to ensure no allocation bias. First, twenty Hospitals selected using the stratified cluster sampling method were randomized into two groups (ten hospitals each) using stratified randomization. Eligibility criteria were considered that may account for potential variations within the clusters that could influence the outcome. Then, the two groups were allocated as the intervention and the control ensuring that each group was comparable at baseline. Hospitals were located in different geographic areas, and only one cluster was selected from each hospital to minimize contamination between study arms. This approach minimized potential biases and ensured a fair distribution of hospitals, enhancing the reliability and internal validity of the study.
  The two steps in randomization were,
  Step I: Twenty hospitals selected through a stratified cluster sampling method were randomized into two groups based on hospital type (Teaching, Special, Base, and Divisional hospitals). Within each stratum, hospitals were randomly allocated to intervention and control arms using the lottery method.
  Step II: Allocation of hospitals to intervention and the control arms was selected by an independent person (Consultant Community Physician attached to MoH), who was not involved in the selection of participants or any other activity of the research study thereby ensuring allocation concealment.
III) Implementation of the intervention - Intervention was conducted without interruption of HCAs’ work in a suitable date, time and place with an appropriate environment after discussing with support from medical and public health staff. Dates and venues were communicated via group-appointed leaders and WhatsApp. The PI assisted by trained RAs, delivered 16 two-hour sessions (Supplementary File 02) over 8 weeks using a facilitator guide, handbook, and PowerPoint, incorporating presentations, interactive discussions, and group activities. A WhatsApp group was used to share educational materials and experiences. Attendance was recorded, dropouts documented, and delivery monitored with process evaluation indicators. To minimize contamination, no personnel, activities, or training materials were shared between clusters during the intervention period. The control group received only routine health services during the study, but was given the BCIP after the post-test with the same interactive format.
IV) Post-assessment - Post-assessment of the level of HL and secondary outcomes among intervention and control arms after two weeks of completion of BCIP. Data collection was conducted using the same tools in both arms through IAQ. Feedback form was also given to the participants in the intervention group.

### Data analysis

The intervention effectiveness was evaluated using primary and secondary outcome indicators, with data entered, cleaned, and analyzed in SPSS version 23.

Participant characteristics including socio-demographics, work-related factors, information sources, language proficiency, and health knowledge were analyzed first. Health knowledge scores ≥ 7/10 were classified as satisfactory, and < 7 as limited based on expert consensus. Categorical variables were compared using Chi-squared or Fisher’s exact tests (independent groups) and McNemar’s test (paired groups).

The primary outcome, HL score and level, was measured using the validated HL-NCD tool. This tool had 56 items on a 4-point Likert scale, giving a total score between 56 and 224. Scores were converted to a percentage, and scores above 50% were considered satisfactory HL. Analysis included within-group comparisons using paired sample t-test and between-group comparisons using independent sample t-test at baseline and after the intervention for HL score. Analysis included within-group comparisons using McNemar’s test and between-group comparisons using the Pearson Chi-squared test at baseline and after the intervention for HL level.

Secondary outcomes were analyzed similarly, with Chi-squared/Fisher’s exact tests for categorical variables and paired/independent t-tests for continuous variables. Behavioural risk factors were assessed using the Sri Lanka STEPS Survey criteria, where the presence of risk behaviour was defined as tobacco or alcohol use within 30 days, less number of varieties of fruit/vegetable intake, adding salt to food, or < 150 min of physical activity per week.

Both unadjusted and cluster-level analyses were conducted to compare baseline and post-intervention outcomes between study arms. To account for intra-cluster correlation, adjusted analyses using Generalized Estimating Equations (GEE) were performed. GEE was selected over mixed-effects modeling as it provides robust population-averaged estimates aligned with the study’s aim of evaluating overall intervention effectiveness without assumptions on random-effect distributions.

**Fig. 1:**
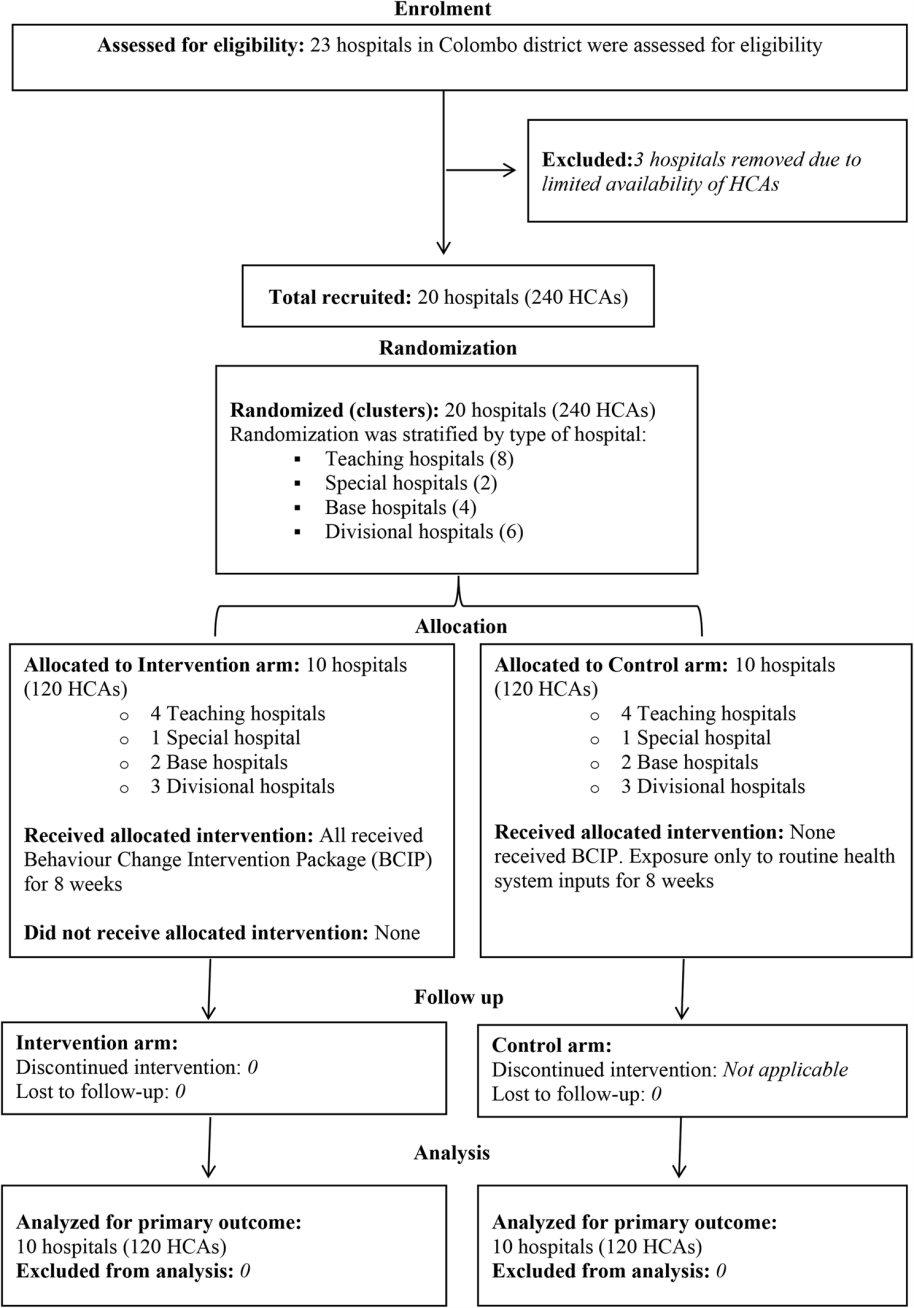
CONSORT flow diagram of the study

Consort Flow diagram of the study is given in Fig. 1.

### Ethical considerations

Approval for the study was obtained from the Board of Study, Community Medicine from the Postgraduate Institute of Medicine, Colombo. Permission from DDG MS II, PDHS of Western province, and RDHS Colombo were obtained. Written permission was obtained from the Directors of each selected hospital. Ethical clearance was obtained from the Ethics Review Committee of the Faculty of Medicine, University of Colombo with the clinical trial registration. (SLCTR/2023/023, https://slctr.lk/trials/slctr-2023-023, 11th December 2023, Sri Lanka Clinical Trial Registry). Informed written consent was obtained from all HCAs, and strict measures for privacy, confidentiality, and data safety were implemented.

## Results

The CONSORT flow diagram detailing participant enrollment, intervention allocation, follow-up, and inclusion in the analysis is given in Fig. 1.

Baseline characteristics of the intervention and control groups are summarized in Table 1. The intervention and control groups were comparable across most socio-demographic and work-related characteristics, with no significant baseline differences except for participation in special health-related training (*p* < 0.001), use of radio (*p* = 0.049) and mass media (*p* = 0.043) for health information, and English proficiency (*p* = 0.005). All participants were proficient in Sinhala, and no difference was found in Tamil proficiency. The GEE models adjusted for baseline covariates and accounted for clustering to estimate intervention effects.

**Table 1:** Comparison of sociodemographic and other basic characteristics of HCAs in the intervention and control groups

Characteristics	Intervention Group (*n* = 120)		Control Group (*n* = 120)		Sig.
	No.	(%)	No.	(%)	
Age in years					*χ2* = 3.774^*^ df = 1 *p* = 0.052
18–35	45	37.5	31	25.8	
36–55	75	62.5	89	74.2	
Sex					*χ2* = 0.153 df = 1 *p* = 0.696
Male	50	41.3	53	44.2	
Female	70	58.3	67	55.8	
Ethnicity ^a^					
Sinhala	118	98.3	119	99.2	*χ2* = 0.338^*^ df = 1 *p* = 1.000
Tamil	2	1.7	0	0.0	
Muslim	0	0.0	1	0.8	
Malay	0	0.0	0	0.0	
Religion ^a^					*χ2* = 0.684^*^ df = 1 *p* = 0.684
Buddhism	116	96.7	118	98.3	
Hinduism	1	0.8	0	0.0	
Christianity	2	1.7	0	0.0	
Roman catholic	1	0.8	1	0.8	
Islam	0	0.0	1	0.8	
Education					*χ2* = 0.826^*^ df = 1 *p* = 0.363
Up to grade 09	3	2.5	10	8.3	
Up to GCE O/L	60	50.0	60	50.0	
Up to GCE A/L	52	43.3	46	38.3	
Above GCE A/L	2	1.7	4	3.3	
Diploma/Degree	3	2.5	0	0.0	
Service years					*χ2* = 0.270 df = 1 *p* = 0.603
Less than 10 years	69	57.5	65	54.2	
More than 10 years	51	42.5	55	45.8	
Participated in special training related to health					*χ2* = 14.423 df = 1 *p* = 0.000
Yes	26	21.7	6	5.0	
No	94	78.3	114	95.0	
Level of Health Knowledge					*χ2* = 1.571 df = 1 *p* = 0.210
Limited	78	65.0	87	72.5	
Satisfactory	42	35.0	33	27.5	
Language Proficiency					
Sinhala	120	100	120	100	Constant
English	35	29.2	17	14.2	*χ*^*2*^ = 7.954 df = 1 *p* = 0.005
Tamil ^a^	3	2.5	2	1.7	*χ*^*2*^ = 0.204 df = 1 *p* = 1.000

*Chi-squared test was considered when rows were amalgamated

^a^Fisher’s exact test was performed since more than 20% of the cells have the expected value < 5

The BCIP produced a significant improvement in HL among HCAs. The median HL score increased from 42.4 to 52.2 in the intervention group (*p* < 0.001, *r* = 0.8), while the control group showed no change (Table 2). The GEE analysis indicated that intervention participants scored 7.8 points higher than controls (95% CI: 7.38–8.22, *p* < 0.001). The proportion achieving satisfactory HL increased significantly (*p* = 0.003) (Table 3), and intervention participants were 3.8 times more likely to reach satisfactory HL than controls (OR = 3.8, 95% CI: 1.59–9.11, *p* = 0.003).

**Table 2:** Comparison of HL score of HCAs in the intervention and control groups

Point in Time	Intervention group (*n* = 120)				Control group (*n* = 120)				Sig* (Betweengroup)	Effect size *r*=(z/√*N*)
	Mean	SD	Median	IQR	Mean	SD	Median	IQR		
Pre- test	42.4	4.7	42.4	4.9	42.7	3.9	42.9	3.6	Z=−1.577 *p* = 0.115	*r* = 0.1
Post- test	50.4	3.8	52.2	4.4	42.7	4.1	42.9	4.5	Z=−10.48 *p* < 0.001	*r* = 0.6
Sig* (Within group)	Z=−9.319 *p* < 0.001				Z=−0.014 *p* = 0.989					
Effect size r=(z/√N)	*r* = 0.8				*r* = 0.0					

**Table 3:** Between group comparison of HL level of HCAs in the intervention and control groups

Level of HL	Pre-test		Sig.	Post-test		Sig.
	Intervention group No. (%)	Control group No. (%)		Intervention group No. (%)	Control group No. (%)	
Limited	108 (90.0)	114 (95.0)	*Χ*^*2*^ *=* 2.162 df = 1 *p* = 0.141	92 (6.7)	111 (92.5)	*χ*^*2*^ *=* 11.535 df = 1 *p* = 0.001
Satisfactory	12 (10.0)	6 (5.0)		28 (93.3)	9 (7.5)	

Significant improvements were also observed in behavioural outcomes. Table 4 summarizes the GEE analysis results. The intervention group showed higher odds of limiting sugar (OR = 12.35, *p* < 0.001) and salt (OR = 9.42, *p* < 0.001) intake, and increasing fruit (OR = 9.15, *p* = 0.026) and vegetable (OR = 5.05, *p* < 0.001) consumption. Adequate physical activity rose from 18.3% to 33.3% (OR = 5.83, *p* < 0.001), and active transport use increased (OR = 5.44, *p* < 0.001). Smokeless tobacco use (OR = 2.70, *p* = 0.022) and exposure to secondhand smoke (OR = 46.72, *p* < 0.001) significantly decreased, while no significant reduction was found in smoking or alcohol use.

**Table 4:** Adjusted analysis for clustering and confounding Variables, using generalized estimating equation with individual data for the effect of the intervention

Outcome	OR	95% CI	*p* value
HL Score	7.801	7.378–8.224	< 0.001
HL level	3.800	1.585–9.113	0.003
Consuming Less than the Recommended Daily amount of Sugar	12.346	4.270–35.701	< 0.001
Consuming Less than the Recommended Daily amount of Salt	9.423	4.538–19.569	< 0.001
Consuming the Recommended Number of Fruits per Day	9.154	1.295–64.717	0.026
Consuming the Recommended Number of Vegetables per Day	5.053	2.466–10.354	< 0.001
Engaging in the Recommended Level of Physical Activity	5.827	3.222–10.537	< 0.001
Using Active Mode of Transport	5.439	2.336–12.663	< 0.001
Not Using Smokeless Tobacco	2.704	1.152–6.350	0.022
Currently Non-Smoking	1.654	0.611–4.478	0.322
Non-exposure to Secondhand smoking	46.718	8.091–269.745	< 0.001
Currently Not Taking Alcohol	1.889	0.401–8.898	0.421

Model: (Threshold), Arm, Age, Sex, Education, Service years, Monthly income, Membership in Health club or Welfare Group, Special Course in Health, Awareness program related to health, Health information from Radio, Health information from mass media, Health information from the internet, Proficiency in English

## Discussion

The BCIP demonstrated robust short-term effectiveness in enhancing both HL scores and HL levels among HCAs, as evidenced by unadjusted, cluster-level, and GEE analyses. The intervention group achieved a large effect size (*r* > 0.8), with median HL scores increasing from 42.4 to 52.2 (*p* = 0.005) and GEE analysis indicating a 7.8-point advantage over controls (OR = 7.801, 95% CI 7.378–8.224, *p* < 0.000). These findings indicate that a structured, theory-driven HL intervention can produce meaningful short-term improvements in HL within an occupational group with important public health responsibilities.

Age < 35 years and higher education were significant positive predictors of HL, consistent with the NAAL survey in the USA and German findings of greater HL and willingness to improve HL among younger adults [17–19], while education only up to O/Ls predicted poorer HL (OR = 0.465, *p* = 0.029),aligning with evidence that identifies low HL as a mediator between lower education and adverse health outcomes [18, 20].

The strong intervention effects are likely attributable to BCIP’s grounding in the Calgary Charter and its emphasis on practical skill-building features that consistently identified in systematic reviews as critical to intervention success [21, 22]. Although the HL-NCD tool assesses four distinct HL domains - finding standard information, understanding self practice, comparing, and applying; domain-specific analyses were not conducted in this study. This was primarily due to the study’s focus on overall HL improvement and sample size considerations that limited statistical power for robust domain-level comparisons. This limits understanding of which HL components contributed most to behaviour improvement. Future studies with larger samples should incorporate domain-specific analyses to better elucidate the pathways through which HL improvements translate into behavioural change.

With regard to behavioural outcomes, the BCIP was associated with significant improvements in diet-related practices, including increased adherence to recommended sugar and salt limits and improved fruit and vegetable intake. These findings are consistent with prior evidence demonstrating that group-based, theory-driven HL interventions can effectively influence dietary behaviours [9, 19, 22]. Physical activity levels also improved substantially following the intervention, supporting evidence from systematic reviews indicating that education- and counselling-based interventions, as well as multi-component workplace programs, can promote physical activity [23–25]. A two-week follow-up period was used to minimize recall bias, consistent with previous behaviour change interventions assessing diet and physical activity [22]. While improvements observed at follow-up indicate immediate post-intervention effects, the sustainability of dietary and physical activity behaviour changes cannot be determined due to the limited follow-up duration. Future research should incorporate extended follow-up periods of 6–12 months to adequately assess the sustainability and long-term impact of the intervention.

For addictive behaviours, the BCIP resulted in significant reduction in smokeless tobacco use (OR = 2.704, *p* = 0.022) and secondhand smoke exposure (OR = 46.718, *p* < 0.000), aligning with prior evidence supporting behavioural interventions for smokeless tobacco cessation [26–28]. In contrast, no significant reductions were observed in smoking tobacco and alcohol use, likely due to the socially reinforced and habitual nature of these behaviours, which require longer-term, individualized, multi-modal approaches and sustained support [28, 29]. This differential responsiveness highlights an important implication for intervention design: while HL-focused, group-based programs may be effective for modifying diet and physical activity, more intensive or specialised strategies such as sustained counselling, brief interventions, pharmacological support, or structured cessation services are likely necessary to address addictive behaviours, even among motivated occupational groups.

Although randomization was stratified by hospital type and analyses adjusted for baseline covariates using GEE models, several baseline imbalances were observed between intervention and control groups. Participants in the intervention group had greater prior exposure to special health-related training, higher use of radio and mass media for health information, and better English proficiency. These factors may have facilitated improved access to information and greater receptivity to HL-related content, potentially contributing to the magnitude of HL improvement observed. While statistical adjustment was applied to mitigate these effects, residual confounding cannot be entirely excluded and should be considered when interpreting the findings.

The reliance on self-reported HL and behavioural data represents another limitation. Although validated Sri Lankan STEPS Survey components were used, self-reported measures are susceptible to social desirability and recall biases, which may have led to an overestimation of positive behavioural changes, particularly for diet and physical activity. Future research should incorporate objective outcome measures, such as biochemical validation for tobacco or alcohol use and device-based monitoring of physical activity, to strengthen the validity of behavioural assessments.

Despite these limitations, the BCIP among a relatively homogenous group of HCAs indicates strong potential for scalability and adaptation to other workplace and community settings. Its modular design and combination of face-to-face and m-Health components enhance feasibility in resource-limited contexts, while its group-based format supports cost-effectiveness through peer learning. Although only one cluster was selected from each hospital and the BCIP content was withheld from the control group until the end of the intervention to minimize contamination, the possibility of limited information exchange between participants cannot be entirely excluded.

Overall, these findings reinforce evidence from low- and middle-income country settings that HL interventions can produce substantial short-term improvements in diet, physical activity, tobacco use, and self-care behaviours [30]. By integrating a theory-driven framework, comprehensive HL measurement, and skill-based learning, the BCIP offers a novel, scalable, and potentially cost-effective model for addressing NCD risk factors through HL enhancement. Future studies incorporating domain-specific HL analyses, objective outcome measures, and longer follow-up periods of 6–12 months are needed to clarify mechanisms of action and assess long-term sustainability.

## Conclusions

The BCIP demonstrated short-term effectiveness in enhancing both HL scores and HL levels among HCAs in Colombo district hospitals. The intervention led to significant improvements in self-reported dietary practices including reduced sugar and salt consumption and increased fruit and vegetable intake as well as notable gains in physical activity and active transport. Reductions in smokeless tobacco use and exposure to secondhand smoke were also observed; however, no significant changes were identified for smoked tobacco or alcohol intake during the study period. These heterogeneous outcomes highlight that while the BCIP effectively promoted healthier lifestyle practices, certain entrenched behaviours may require longer-term or more intensive strategies.

Grounded in the four domains of HL, the BCIP demonstrates the potential of HL-based approaches to strengthen individual capacity for informed health decisions and self-management. Integrating such HL-focused programs into routine staff training or national NCD control initiatives could enhance their reach and sustainability. Overall, the BCIP contributed to improved self-reported behavioural practices and strengthened HL among healthcare workers, offering a promising and scalable approach to support NCD prevention in similar resource-limited contexts.

## Supplementary Information

Supplementary Material 1.

Supplementary Material 2.

## Data Availability

The datasets generated and analyzed during the current study are not publicly available due to participant confidentiality and ethical restrictions, but may be available from the corresponding author on reasonable request.

## Abbreviations

FFQ: Food Frequency Questionnaire
GPAQ: Global Physical Activity Questionnaire
HL: Health Literacy
HCA: Health Care Assistant
HL-NCD: Health Literacy on Behavioural risk factors of NCDs
NCD: Non Communicable Disease
