# Supplementary material for: Effectiveness of a behaviour change intervention on health literacy for behavioural risk factors of non-communicable diseases among health care assistants of government hospitals in Colombo District: a cluster-randomized controlled trial
Source: BMC Public Health. 2026 Jan 2;26:421. doi: 10.1186/s12889-025-26110-9 (PMC12866114; doi:10.1186/s12889-025-26110-9)
Supplement: Supplementary file 1 — Supplementary Material 1. [file 12889_2025_26110_MOESM1_ESM.pdf]

HL-NCD Questionnaire

Interviewer-administered questionnaire to assess health literacy on behavioral risk factors of NCD and the associated factors.

Please mark 'X' in the relevant box or write the correct answer in the given space.

<u>Section – A – General Information</u>

A1. What is your age as at your last birthday? _ _ _ years

A2. Your sex

1. Male ☐ 2. Female ☐

A3. Your Ethnicity

1. Sinhala ☐
2. Tamil ☐
3. Muslim ☐
4. Burgher ☐
5. Other (please specify) ☐

A4. Your Religion

1. Buddhism ☐
2. Hindu ☐
3. Roman Catholic ☐
4. Christian ☐
5. Islam ☐
6. Other ☐

A5. What is your highest educational qualification?

1. Up to grade 09 ☐
2. Up to GCE O/L ☐
3. Up to GCE A/L ☐
4. above GCE A/L ☐
5. Diploma/Degree ☐

A6. How long have you been in the service as Health Care Assistant?

--------------------- Years --------------------- Months

A7. Your marital status

1. Married ☐
2. Unmarried ☐
3. Widowed ☐
4. Divorced ☐

If unmarried go to 13

A8. Do you have children?

1. Yes ☐ 2. No ☐

If "no", go to question 10

A9. If yes, how many children do you have? -------------

A10. What is your spouse's highest educational achievement?

1. No schooling ☐
2. Up to Grade 5 ☐
3. Grade 6-9 ☐
4. Up to G.C.E (O/L) ☐
5. Up to G.C.E (A/L) ☐
6. University education and above ☐

A11. What is your spouse's employment status?

1. Currently employed ☐
2. Previously employed ☐
3. Never employed ☐

A12. Is your spouse suffering from a condition that requires long term medical care?

1. Yes ☐ 2. No ☐

A13. Do you have someone who is engaged in a health-related profession (E.g: - doctor, nurse,) in your immediate family?

1. Yes ☐ 2. No ☐

If "no", go to question 15

A14. Indicate the member and the profession?

1. Spouse ☐ Profession -----------------
2. Mother/Father ☐ Profession -----------------
3. Siblings ☐ Profession -----------------
4. Children ☐ Profession -----------------

A15. What is your monthly household income?

(Please state the average income of your household. It means the money you get for a month from one or more different sources such as your salary from the job, interest of deposits/ loans given to others, vehicles/buildings you have rented to others, business shares, income from loans etc. This includes the overall earnings from all members of the household. When you give us the amount, add all these income sources and estimate an average amount for a month.)

1. Less Than Rs.25000
2. More Than Rs.25000 – Rs.50000
3. More Than Rs.50000- Rs.75000
4. More Than Rs.75000

A16. Are you a member of health club/welfare group in your community during last six months?

1. Yes ☐ 2. No ☐

Exposure to the health education or special training of the participant

A17.Have you done any special course in health-related subjects during last six months?

1. Yes ☐ 2. No ☐

A18. Within last six months have you ever got an opportunity to participate in an awareness program related to health (E.g.: - Listening to a lecture on Nutrition, Attending a workshop)?

1. Yes ☐ 2. No ☐

Self-Assessed Health Status and contact with health care professionals by the participant

A19. Have you been diagnosed with any of the following conditions?
Mark all relevant cages with “X”

1. Diabetes ☐
2. Hypertension ☐
3. Malignant disease ☐
4. Ischemic heart disease ☐
5. Chronic renal disease ☐
6. Asthma/COPD ☐
7. Arthritis ☐
8. Thalassemia ☐
9. Thyroid disease ☐
10. Other (Specify)………….. ☐
11. No ☐

A20. During last six months, how frequently did you obtain advice from a medical practitioner in a private clinic/ channel service/ OPD of a government hospital or met with a member of the preventive health staff such as Medical Office of Health, Public Health Midwife or Public Health Inspector to obtain medical treatment/ health advice for you or anybody else?

1. Not contacted ☐

If contacted, number of times................

A21.Have any of your immediate family members (siblings, parents) ever been diagnosed with any of the following conditions?
Mark all relevant cages with "X"

1. Diabetes ☐
2. Hypertension ☐
3. Malignant disease ☐
4. Ischemic heart disease ☐
5. Chronic renal disease ☐
6. Nephrotic/ Nephritic syndrome ☐
7. Congenital disorders- Cerebral Palsy/ Downs syndrome ☐
8. Blood disorders – Thalassemia/ Hemophilia ☐
9. Asthma/COPD ☐
10. Thyroid disease ☐
11. Arthritis ☐
12. Drug abuse ☐
13. Other (Specify) ------------------- ☐

Access and usage of health communication channels

A22. During the past six months, which of the following mass media modes were used by you to obtain **general** information (e.g. news, weather etc.)?

1.	TV	Yes	☐	No	☐
2.	Radio	Yes	☐	No	☐
3.	Print media (Newspapers, Magazines)	Yes	☐	No	☐
4.	Mass media	Yes	☐	No	☐
5.	Internet	Yes	☐	No	☐

If "no" to all, go to question 24

A23. During the past six months, which of the following mass media modes were used by you to obtain **health** information?

1.	TV	Yes	☐	No	☐
2.	Radio	Yes	☐	No	☐
3.	Print media (Newspapers, Magazines)	Yes	☐	No	☐
4.	Mass media	Yes	☐	No	☐
5.	Internet	Yes	☐	No	☐

If “no” to all, go to question 24

Language ability

A24. What are the languages that you can speak?

Mark all relevant cages with “X”

1. Sinhala Yes ☐ No ☐
2. English Yes ☐ No ☐
3. Tamil Yes ☐ No ☐

A25. What are the languages that you can read?

Mark all relevant cages with “X”

1. Sinhala Yes ☐ No ☐
2. English Yes ☐ No ☐
3. Tamil Yes ☐ No ☐

A26. What are the languages that you can write?

Mark all relevant cages with “X”

4. Sinhala Yes ☐ No ☐
5. English Yes ☐ No ☐
6. Tamil Yes ☐ No ☐

Health Knowledge

Following questions from A27 to A36 are single best response type questions. Select only one correct answer out of the 5 responses.

A27. What is the commonest Non-Communicable Disease in Sri Lanka?

1. Diabetes Mellitus ☐
2. Dengue ☐
3. Tuberculosis ☐
4. Leprosy ☐
5. Pneumonia ☐

A28. Which of the following is a common intermediate risk factor of common non-communicable diseases in Sri Lanka?

1. Infection ☐
2. High BMI (Obesity) ☐
3. Fever ☐
4. Injury ☐
5. Allergic rhinitis ☐

A29. Which of the following is common modifiable (Changeable) risk factor for Non-Communicable Disease in Sri Lanka?

Lack of hygiene ☐

Using multimedia ☐

Unhealthy diet ☐

Unsafe sex ☐

High speed driving ☐

A30. Which of the following foods is rich in carbohydrate?

1. Rice ☐
2. Fruits ☐
3. Green Leaves ☐
4. Fish ☐
5. Vegetables ☐

A31. Which of the following statement is correct?

1. Smoking in low numbers per day is safe. ☐
2. Smoking of branded cigarettes is not harmful to the body. ☐
3. Physical activity reduces the risk of developing Non-Communicable Diseases. ☐
4. Alcohol can be consumed occasionally without any harmful effects. ☐
5. Excessive carbohydrate intake is good for our health. ☐

A32. What is the recommended (minimum) level of physical activity for a healthy **adult** per day for him/her to stay healthy?

a. 30 minutes of moderate (e.g. brisk walk, aerobic exercises, cycling) Physical activity per day for at least 3 days a week ☐
b. 30 minutes of moderate (e.g. brisk walk, aerobic exercises, cycling) ☐
c. Physical activity per day for at least 5 days a week ☐
d. 1 hour of moderate (e.g. brisk walk, aerobic exercises, cycling) Physical activity per day for at least 5 days a week ☐
e. 2 hours of moderate (e.g. brisk walk, aerobic exercises, cycling) ☐

A33. How many varieties of fruits and vegetables is recommended to be eaten by a healthy person per day for him/her to stay healthy?

1. 1-2 fruits and vegetables a day ☐
2. 2-3 fruits and vegetables a day ☐
3. 5 fruits and vegetables a day ☐
4. 7 fruits and vegetables a day ☐
5. 10 fruits and vegetables a day ☐

A34. What is the ideal BMI value for a healthy person?

1. Less than 18.5 Kg/m^2 ☐
2. Between 18.5 - 23 Kg/m^2 ☐
3. More than 25 Kg/m^2 ☐
4. More than 27 Kg/m^2 ☐
5. More than 30 Kg/m^2 ☐

A35. Which of the following is not an important component of food label?

1. Sugar level ☐
2. Date of Expiry ☐
3. Salt level ☐
4. Size of the pack ☐
5. Fat level ☐

A36. Which of the following statement is correct?

1. Betel chewing does not cause cancer. ☐
2. It is not harmful to inhale the smoke released by a cigarette smoker. ☐
3. Cigarettes are allowed to be sold to individuals less than 18 years in Sri Lanka. ☐
4. Screening for Non-Communicable Diseases is available in Sri Lanka. ☐
5. Drinking branded Alcohol is not harmful for the body. ☐

Section B - Health Literacy Questionnaire (HL-NCD)

Please choose the scale from Never to Always how often you engage in the following activities.

1. **Never** – Not at all
2. **Sometimes** – Minimum once in life
3. **Often** – Minimum once per year
4. **Always** – Minimum once per quarter

1. How frequently you find the current recommended information related to following activity?

No	Activity	Never	Sometimes	Often	Always
1.	How frequently do you find current recommended information regarding total sugar intake including food with sugar?	Never	Sometimes	Often	Always
2.	How frequently do you find current recommended information regarding total salt intake including food with salt?	Never	Sometimes	Often	Always
3.	How frequently do you find current recommended information regarding the daily fat intake including food with fat?	Never	Sometimes	Often	Always
4.	How frequently do you find current recommended information regarding the number of servings of fruits intake on average per day?	Never	Sometimes	Often	Always
5.	How frequently do you find current recommended information regarding the number of servings of vegetables intake on average per day?	Never	Sometimes	Often	Always
6.	How frequently do you find current recommended information regarding the amount of water intake (in liters)?	Never	Sometimes	Often	Always
7.	How frequently do you find current recommended information regarding the content of food on your meal plate including type of food?	Never	Sometimes	Often	Always
8.	How frequently do you find current recommended information regarding the content on food labels of packeted food you purchase?	Never	Sometimes	Often	Always
9.	How frequently do you find current	Never	Sometimes	Often	Always

	recommended information regarding the level of physical activity in your routine life?				
10.	How frequently do you find current recommended information regarding the modes of transport to and from working place/shopping?	Never	Sometimes	Often	Always
11.	How frequently do you find current recommended information regarding the smokeless tobacco use (Eg: Chewing betel with tobacco)?	Never	Sometimes	Often	Always
12.	How frequently do you find current recommended information regarding the tobacco use as smoking?	Never	Sometimes	Often	Always
13.	How frequently do you find current recommended information regarding the secondhand smoking?	Never	Sometimes	Often	Always
14.	How frequently do you find current recommended information regarding the alcohol intake?	Never	Sometimes	Often	Always

2. **How frequently you understand the level of following activity in your routine life?**

No.	Activity	Never	Sometimes	Often	Always
1.	How frequently do you understand your total daily sugar intake including food with sugar?	Never	Sometimes	Often	Always
2.	How frequently do you understand your total daily salt intake including food with salt?	Never	Sometimes	Often	Always
3.	How frequently do you understand your total daily fat intake including food with fat?	Never	Sometimes	Often	Always
4.	How frequently do you understand your number of servings of fruits intake on average per day?	Never	Sometimes	Often	Always
5.	How frequently do you understand your number of servings of vegetables intake on average per day?	Never	Sometimes	Often	Always
6.	How frequently do you understand your amount of water intake (in liters)?	Never	Sometimes	Often	Always
7.	How frequently do you understand content of food on your meal plate including type of food?	Never	Sometimes	Often	Always
8.	How frequently do you understand content on food labels of packeted food you purchase?	Never	Sometimes	Often	Always
9.	How frequently do you understand the level of physical activity in your routine life?	Never	Sometimes	Often	Always
10.	How frequently do you understand the modes of transport to and from working place/shopping?	Never	Sometimes	Often	Always
11.	How frequently do you understand the status of your smokeless tobacco use (Eg: Chewing betel with tobacco)?	Never	Sometimes	Often	Always
12.	How frequently do you understand the status of your tobacco use as smoking?	Never	Sometimes	Often	Always
13.	How frequently do you understand your status of secondhand smoking	Never	Sometimes	Often	Always
14.	How frequently do you understand the status of your alcohol intake?	Never	Sometimes	Often	Always

3. **How frequently you compare level of activity in your routine life with the recommended level of activity?**

No.	Activity	Never	Sometimes	Often	Always
1.	How frequently do you compare the sugar intake with current recommended level?	Never	Sometimes	Often	Always
2.	How frequently do you compare the salt intake with current recommended level?	Never	Sometimes	Often	Always
3.	How frequently do you compare the daily fat intake including food with fat with current recommended level?	Never	Sometimes	Often	Always
4.	How frequently do you compare the number of servings of fruits intake on average per day with current recommended level?	Never	Sometimes	Often	Always
5.	How frequently do you compare the number of servings of vegetables intake on average per day with current recommended level?	Never	Sometimes	Often	Always
6.	How frequently do you compare the amount of water intake (in liters) with current recommended level?	Never	Sometimes	Often	Always
7.	How frequently do you compare the content of food on your meal plate including type of food with current recommended level?	Never	Sometimes	Often	Always
8.	How frequently do you compare the content on food labels of packeted food you purchase with current recommended level?	Never	Sometimes	Often	Always
9.	How frequently do you compare the level of physical activity in your routine life with current recommended level?	Never	Sometimes	Often	Always
10.	How frequently do you compare the modes of transport to and from working place/shopping with current recommended level?	Never	Sometimes	Often	Always
11.	How frequently do you compare the status of smokeless tobacco use (eg: Chewing betel with tobacco) with current recommended level?	Never	Sometimes	Often	Always
12.	How frequently do you compare the status of tobacco use as smoking with current recommended level?	Never	Sometimes	Often	Always
13.	How frequently do you compare the status of secondhand smoking with current recommended level?	Never	Sometimes	Often	Always
14.	How frequently do you compare the status of	Never	Sometimes	Often	Always

	alcohol intake with current recommended level?				

4. **How frequently you apply the recommended level of activity in your routine life?**

No.	Activity	Never	Sometimes	Often	Always
1.	How frequently do you apply the current recommended level of sugar intake?	Never	Sometimes	Often	Always
2.	How frequently do you apply the current recommended level of salt intake?	Never	Sometimes	Often	Always
3.	How frequently do you apply the current recommended level of daily fat intake including food with fat?	Never	Sometimes	Often	Always
4.	How frequently do you apply the current recommended level of number of servings of fruits intake on average per day?	Never	Sometimes	Often	Always
5.	How frequently do you apply the current recommended level of number of servings of vegetables intake on average per day?	Never	Sometimes	Often	Always
6.	How frequently do you apply the current recommended amount of water intake (in liters)?	Never	Sometimes	Often	Always
7.	How frequently do you apply the current recommended level of content of food on your meal plate including type of food?	Never	Sometimes	Often	Always
8.	How frequently do you apply the current recommendation on food label of packeted food when you purchase?	Never	Sometimes	Often	Always
9.	How frequently do you apply the current recommended level of physical activity in your routine life?	Never	Sometimes	Often	Always
10.	How frequently do you apply the current recommended level of modes of transport to and from working place/shopping in you routine life?	Never	Sometimes	Often	Always
11.	How frequently do you apply the current recommended level of smokeless tobacco use in your routine life? (eg: Chewing betel with tobacco)	Never	Sometimes	Often	Always
12.	How frequently do you apply the current recommended level of tobacco use as smoking in your routine life?	Never	Sometimes	Often	Always

13.	How frequently do you apply the current recommended level of exposure to secondhand smoking in your routine life?	Never	Sometimes	Often	Always
14.	How frequently do you apply the current recommended level of alcohol intake in your routine life?	Never	Sometimes	Often	Always

-- --

Date Data Collector's Signature
