# Supplementary material for: Effectiveness of a behaviour change intervention on health literacy for behavioural risk factors of non-communicable diseases among health care assistants of government hospitals in Colombo District: a cluster-randomized controlled trial
Source: BMC Public Health. 2026 Jan 2;26:421. doi: 10.1186/s12889-025-26110-9 (PMC12866114; doi:10.1186/s12889-025-26110-9)
Supplement: Supplementary file 2 — Supplementary Material 2. [file 12889_2025_26110_MOESM2_ESM.pdf]

Supplementary File 02: Outline of the BCIP Curriculum

Session	Topic	Key Learning Outcomes
1	Health Literacy – Concept, Definition & Four Domains	Understand HL concept (Calgary Charter), definition, four domains, and link between limited HL and health status
2	HL Domain 1 – Finding Recommended Health Information	Identify and locate reliable health information, apply in practical scenarios
3	HL Domain 2 – Understanding Daily/Routine Practice	Recognize and interpret routine practices in the context of health
4	HL Domain 3 – Comparing Practice vs. Recommendations	Compare current practices with recommendations, identify gaps
5	HL Domain 4 – Applying Recommendations in Daily Practice	Apply recommended health information effectively in daily life
6	Unhealthy Diet I – Salt, Sugar, Fat & Food Labels	Understand SSF risks, read labels, identify healthy diet components, know daily SSF limits
7	Unhealthy Diet II – Fruits, Vegetables, Water, Healthy Plate	Identify unhealthy plates, apply recommended intake of fruits, vegetables, and water
8	Physical Inactivity	Understand risks, barriers, and interventions for physical inactivity; prescribe suitable activity
9	Tobacco Use	Understand health & environmental harms, reasons for use, legal context, and quitting strategies
10	Alcohol Intake	Recognize health risks, alcohol types, related problems, harms to others, and interventions

11	Behaviour Change – Diet (HL 4 Domains)	Find, understand, compare, and apply healthy diet recommendations
12	Behaviour Change – Physical Activity (HL 4 Domains)	Find, understand, compare, and apply physical activity recommendations
13	Behaviour Change – Tobacco Use (HL 4 Domains)	Find, understand, compare, and apply tobacco-related recommendations (smoke & smokeless)
14	Behaviour Change – Alcohol Intake (HL 4 Domains)	Find, understand, compare, and apply alcohol-related recommendations
15	Sustainability of Behaviour Change (Mind–Body–Memory)	Link mind, memory, and body; maintain healthy habits; reinforce behaviour change
16	Applying HL to Other Health Aspects	Use HL skills to find, understand, compare, and apply recommendations in various health areas
